# Blockage-Aware Power Allocation Algorithm for Millimeter-Wave Communication with Dynamic Reward Q-Learning

**DOI:** 10.3390/s26154872

**Published:** 2026-08-02

**Authors:** Zhuoning Yang, Ziwei Chen

**Affiliations:** 1 Faculty of Science and Engineering, The University of Manchester, Oxford Rd., Manchester M13 9PL, UK; 2 Department of Electronics, Beijing Jiaotong University, Beijing 100044, China

**Keywords:** blockage-aware power control, distributed Q-learning, millimeter-wave communication, state-dependent reward, LOS/NLOS transition

## Abstract

Millimeter-wave (mmWave) communication systems are vulnerable to severe attenuation, blockage-induced LOS/NLOS transitions, and time-varying co-channel interference. This paper develops a lightweight distributed power-allocation framework in which each base station independently updates a tabular Q-learning policy using locally observable blockage-ratio, serving-distance, and aggregate-interference information. The proposed state-dependent dynamic reward is recalculated at every decision step, and its coefficients vary explicitly with the instantaneous blockage ratio, QoS satisfaction ratio, and normalized interference level. All learning-based and non-learning baselines are evaluated using the same topology realizations, blockage and mobility traces, and random seeds. Under the reconstructed simulation settings, the proposed method achieves performance comparable to fixed Q-learning while retaining a transparent blockage-aware state and low-complexity distributed implementation. DQN, greedy, and uniform power achieve higher raw capacity or QoS in the considered small-scale network. Results from 30 paired runs with 95% confidence intervals, together with ablation, sensitivity, beam-misalignment, and overhead analyses, clarify the empirical benefits, limitations, and deployment scope of the proposed method. The study focuses on power control after beam establishment; joint beam tracking and power allocation remain outside the present scope.

## 1. Introduction

The rapid evolution of 5G and beyond-5G wireless systems has increased the demand for high data rates, low latency, and dense connectivity [1]. Millimeter-wave (mmWave) communication is regarded as a key enabling technology because it can exploit large bandwidth resources and support high-capacity wireless transmission [2,3]. However, mmWave links are highly sensitive to propagation loss, blockage, and line-of-sight/non-line-of-sight (LOS/NLOS) transitions. Measurement-based channel studies, stochastic-geometry analyses, and the 3GPP channel model all show that distance-dependent LOS/NLOS propagation and blockage have a direct impact on coverage, rate, and link reliability [4,5]. These characteristics make power allocation in mmWave networks more challenging than in conventional sub-6 GHz systems, especially in dense deployments and device-to-device communication scenarios [6].

Power allocation is an important mechanism for balancing system capacity, QoS satisfaction, and interference control. Centralized optimization methods can provide effective solutions when global channel state information and network-wide interference information are available. However, in ultra-dense and dynamically changing mmWave networks, collecting global information may introduce substantial signaling overhead, computational complexity, and control delay. Existing surveys on wireless resource allocation also indicate that scalable and low-overhead resource-management strategies are essential for heterogeneous and dense wireless networks [7]. Therefore, distributed power-control mechanisms that rely on locally observable information are more suitable for practical deployment when the radio environment changes rapidly due to blockage and mobility.

Reinforcement learning (RL) has been widely investigated for adaptive wireless resource allocation because it can learn decision policies through interaction with uncertain environments. Q-learning provides a lightweight model-free framework for discrete state-action spaces [8], while deep reinforcement learning methods such as deep Q-network (DQN) provide stronger function approximation capability for large-scale state spaces [9,10]. In wireless communication systems, DRL has been used for joint beamforming, power control, and interference coordination in 5G/mmWave networks [11], and learning-based mechanisms have also been studied for dynamic blockage mitigation and wireless learning/communication co-design [12,13]. Transfer Q-learning has further been investigated for 5G New Radio mmWave networks, indicating the relevance of lightweight RL mechanisms in mmWave resource-management problems [14].

Despite these advances, several issues remain insufficiently addressed in lightweight distributed power allocation for blockage-varying mmWave networks. First, many existing learning-based methods use generic channel-quality or interference indicators, whereas the LOS/NLOS condition is not explicitly represented as a local state variable for power-control decisions. Second, reward functions are commonly defined using a single throughput objective or a fixed composite objective without clearly explaining how instantaneous blockage, QoS, interference, and power consumption jointly determine the feedback received by an agent. Third, DQN-based methods provide strong function approximation but require neural-network training, replay memory, target-network updates, and additional hyperparameter tuning; these costs may be unnecessary when the relevant state-action space can be represented by a compact table. Fourth, independent multi-agent learning introduces non-stationarity because each base station changes the interference environment experienced by the others. Finally, many power-control studies abstract beam management through effective antenna gains, and the resulting scope and limitations should be stated explicitly. These gaps motivate a reproducible distributed tabular framework with an explicit blockage-aware state, clearly defined state-dependent reward feedback, and transparent deployment assumptions.

Motivated by the above considerations, this paper proposes a distributed blockage-aware power allocation framework based on dynamic reward Q-learning for mmWave communication networks. In the proposed framework, each base station acts as an independent learning agent and adjusts its transmit power according to locally observable blockage state, user-distance level, and interference-intensity level. A dynamic reward mechanism is further designed to guide the learning process by jointly considering local capacity, QoS satisfaction, interference suppression, transmit-power regularization, and blockage-related penalties. The objective is not to introduce a completely new reinforcement learning paradigm, but to develop a reproducible and low-complexity learning framework tailored to blockage-varying mmWave power control. The main contributions of this paper are summarized as follows:

1. Blockage-aware local state representation for mmWave power control. The local state observed by each base station explicitly incorporates the LOS/NLOS blockage condition, user-distance level, and interference-intensity level, enabling the learning agent to capture blockage-induced channel variations during power-control decisions.
2. State-dependent dynamic reward for environment-aware learning. The immediate reward is recalculated at every decision step from the current normalized throughput, QoS satisfaction ratio, aggregate interference, transmit power, and blockage ratio. Unlike a fixed-weight composite reward, the capacity, QoS, interference, and blockage coefficients are adjusted explicitly according to the current local condition. The update equations and coefficient values are provided in Section 3.2, making the term dynamic reward mathematically reproducible rather than merely descriptive.
3. Distributed Q-learning implementation with local observations. Each base station maintains and updates its own Q-table using local state transitions and local reward feedback. Online decision-making does not require a centralized controller or global channel state information, while interactions among base stations are reflected through the coupled interference caused by their power-control actions.
4. Comprehensive validation against learning-based and non-learning baselines. The proposed method is compared with fixed Q-learning, DQN-based power allocation, greedy power allocation, and uniform power allocation under the same simulation settings. The evaluation includes capacity comparison, convergence behavior, QoS satisfaction analysis, ablation study, robustness analysis, and parameter sensitivity analysis based on 30 independent runs and 95% confidence intervals.

The remainder of this paper is organized as follows. Section 2 presents the mmWave system model and problem formulation. Section 3 describes the proposed distributed dynamic reward Q-learning framework and the DQN-based comparison baseline. Section 4 reports the simulation setup and performance evaluation. Section 5 concludes the paper and discusses future research directions.

## 2. System Model and Problem Formulation

**Figure 1:**
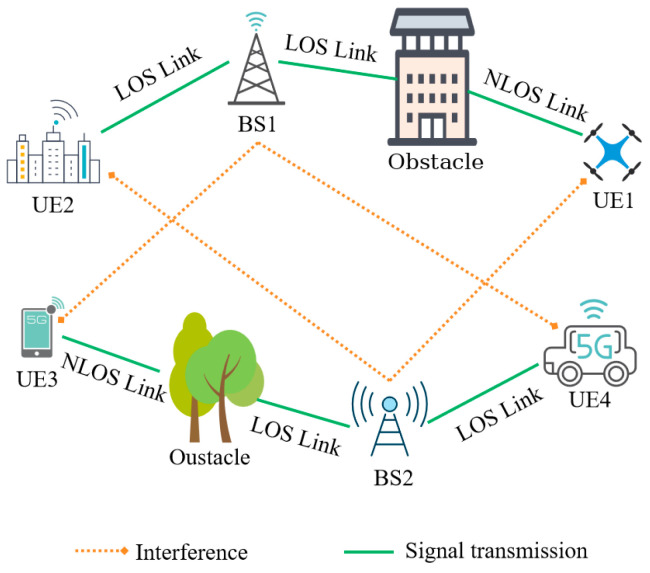
System model of the considered mmWave communication network.

Figure 1 illustrates the considered mmWave communication network, which consists of multiple base stations (BSs), user equipment (UEs), blockage objects, and co-channel interference links. The user locations are modeled using a Poisson cluster process. Each BS–UE link can be in either a line-of-sight (LOS) or non-line-of-sight (NLOS) condition, and the corresponding blockage state affects path loss, SINR, and power-allocation decisions [4,5].

To describe the resource-optimization problem in dynamic mmWave communication networks, this section first presents the network model, blockage-state representation, channel model, and SINR calculation. The resulting model provides the basis for defining the local state, reward design, and distributed power-control objective used in the subsequent reinforcement learning framework.

### 2.1. System Model

The considered network consists of multiple mmWave BSs and UEs. The spatial distribution of users is modeled by a Poisson cluster process to reflect hotspot-like user clustering in dense urban deployments. In this model, the parent point process represents cluster centers, while the daughter points represent users distributed around the corresponding cluster centers. This spatial model captures the non-uniform user distribution commonly observed in dense mmWave networks [4].

User association and update order. Each UE is associated with the BS corresponding to its generating Poisson cluster at the beginning of an episode, and this association is kept fixed within that episode. UE positions may change according to the mobility model, but handover and cell reselection are not considered. At each decision step, all BS agents first observe their local states and select actions; the selected power changes are then applied synchronously. The environment subsequently recalculates the coupled interference, SINR, reward, and next state for every agent. This synchronized procedure prevents an artificial ordering advantage among BSs.

Local observation of inter-BS interference. For UE k served by BS i, co-channel interference is the aggregate received power from all BSs j ≠ i. An agent does not observe neighboring Q-tables or individual actions. Instead, the effect of the other agents is captured implicitly through the aggregate interference measured by its associated UEs and fed back as a discretized local interference level. Consequently, the online policy remains distributed even though the agents are physically coupled through interference.

Directional-antenna scope. The present work studies power control after beam establishment. A serving BS–UE pair is assumed to maintain beam alignment during one power-control decision interval, and the antenna terms in the effective channel gain represent the corresponding effective aligned gains. Beam training, beam switching, array-pattern optimization, and handover are assumed to operate on a separate control timescale. The fixed-gain abstraction does not capture rapid beam misalignment or detailed sidelobe interactions; this limitation is discussed in Section 5.

MmWave communication is highly sensitive to obstacles, and the LOS/NLOS condition is a key factor affecting the channel quality. For the link between BS i and user k, the blockage-state variable is defined as

(1) $$b_{i,k}(t)=\left\{\begin{array}{ll} 1, & \mathrm{LOS},\\ 0, & \mathrm{NLOS}. \end{array}\right.$$

The LOS probability is modeled as a distance-dependent function following the adopted 3GPP urban-microcell mmWave channel model [5]:

(2) $$P_{\mathrm{LOS}}(d_{i,k})=\min\left(\frac{d_{0}}{d_{i,k}},1\right)\left(1-\exp\left(-\frac{d_{i,k}}{d_{c}}\right)\right)+\exp\left(-\frac{d_{i,k}}{d_{c}}\right),\;d_{i,k}>0,$$

where $d_{i,k}$ denotes the distance between BS *i* and user *k*, and $d_{0}$ and $d_{c}$ are environment-dependent parameters determined by the adopted 3GPP mmWave channel scenario.

Temporal LOS/NLOS evolution. The distance-dependent LOS probability is used to initialize each link state. During an episode, the link evolves according to a two-state Markov process with LOS-to-NLOS transition probability p_LN_ and NLOS-to-LOS transition probability p_NL_. After every transition update, the LOS or NLOS path-loss expression is selected accordingly. The updated link states therefore affect the effective channel gains, aggregate interference, SINR values, BS-level blockage state, and blockage-related reward term at the same decision step. The transition probabilities used in the default and robustness experiments are reported in Table 1.

For a BS–user distance, the path loss is represented separately for LOS and NLOS links according to the adopted mmWave channel and blockage model as

(3) $$L_{i,k}^{\mathrm{LOS}}(d_{i,k})=L_{0}+10\alpha_{\mathrm{LOS}}\log_{10}(d_{i,k})+X_{\sigma ,\mathrm{LOS}},$$

(4) $$L_{i,k}^{\mathrm{NLOS}}(d_{i,k})=L_{0}+10\alpha_{\mathrm{NLOS}}\log_{10}(d_{i,k})+X_{\sigma ,\mathrm{NLOS}}.$$

where $L_{0}$ is the reference path loss, $\alpha_{\mathrm{LOS}}$ and $\alpha_{\mathrm{NLOS}}$ denote the path-loss exponents under LOS and NLOS conditions, respectively, and $X_{\sigma ,\mathrm{LOS}}$ and $X_{\sigma ,\mathrm{NLOS}}$ denote the corresponding log-normal shadow-fading terms. Different parameter sets are used for LOS and NLOS links according to the adopted mmWave channel model.

### 2.2. Problem Formulation

Let $P_{i}\left(t\right)$ denote the transmit power of BS i at time step t, and let $U_{i}$ denote the set of users served by BS i. The SINR of user $k\in U_{i}$ is expressed as

(5) $${\mathrm{SIN}R}_{i,k}(t)=\frac{P_{i}(t)G_{i,k}(t)}{\sum_{j\neq i}P_{j}(t)G_{j,k}(t)+\sigma^{2}},$$

$$G_{i,k}\left(t\right)\sigma^{2}G_{i,k}\left(t\right)=G_{i}^{Tx}G_{k}^{Rx}10^{-L_{i,k}\left(t\right)/10},G_{i}^{Tx}G_{k}^{Rx}L_{i,k}\left(t\right)b_{i,k}\left(t\right)b_{i,k}\left(t\right)=1b_{i,k}\left(t\right)=0$$

where the effective channel gain includes the selected LOS/NLOS path loss and the effective antenna gains, and the noise term denotes the receiver noise power. The denominator includes co-channel interference from all non-serving BSs. Thus, although BS i updates its policy using local information, the action of every other BS affects its reward and next state through the coupled interference term. For the desired link, the LOS model is used when the current blockage state is LOS and the NLOS model is used otherwise.

The achievable rate of user k served by BS i is given by

(6) $$C_{i,k}(t)=B\log_{2}\left(1+{\mathrm{SINR}}_{i,k}(t)\right),$$

where B denotes the system bandwidth. The total system capacity is then given by

(7) $$C_{\mathrm{tot}}(t)=\sum_{i}\sum_{k\in U_{i}}C_{i,k}(t).$$

The transmit power of each BS is limited by the transmit-power constraint:

(8) $$P_{min}\leq P_{i}\left(t\right)\leq P_{max},\;\forall i,t$$

where $P_{i}\left(t\right)$ denotes the linear transmit power in watts. The lower and upper linear power limits are converted from the dBm-scale limits as $P_{min}=10^{\left(p_{min}-30\right)/10}$ and $P_{max}=10^{\left(p_{max}-30\right)/10}$, respectively. The SINR threshold is specified in dB as $\gamma_{th}^{dB}$, while its linear-scale value $\gamma_{th}^{lin}=10^{\gamma_{th}^{dB}/10}$ is used in the QoS evaluation and reward calculation. Due to blockage-induced channel degradation, not all users can always satisfy this threshold under severe blockage conditions. Therefore, the QoS satisfaction rate is reported as a reliability-oriented performance metric:

(9) $$\rho_{QoS}\left(t\right)=\frac{\sum_{i}\sum_{k\in U_{i}}q_{i,k}^{QoS}(t)}{\sum_{i}\left|U_{i}\right|}$$

where $q_{i,k}^{QoS}(t)$ denotes a binary QoS satisfaction indicator. It equals one when ${SINR}_{i,k}(t)\geq \gamma_{th}^{lin}$ and equals zero otherwise. This formulation avoids treating the QoS threshold as an always-feasible hard constraint and is consistent with the severe blockage cases evaluated in the Section 4.

In centralized power control, a controller typically collects network-wide channel, association, and interference information before computing a joint power vector. In the distributed formulation adopted here, each BS uses only its local blockage summary, average serving-distance level, and measured aggregate-interference level. The agents do not exchange Q-tables or intended actions. Their interaction is nevertheless represented through the physical interference coupling in the SINR calculation. User association is fixed within each episode, and all BS actions are applied synchronously before the next-state and reward calculations.

Accordingly, the power-allocation objective is formulated as maximizing the total system capacity under the transmit-power boundary:

(10) $$\underset{\left\{P_{i}(t)\right\}}{\max}C_{\mathrm{tot}}(t)$$

(11) $$s.t.\;P_{min}\leq P_{i}\left(t\right)\leq P_{max},\;\forall i,t.$$

The QoS satisfaction rate defined above is then incorporated into the reward design and used as an evaluation metric. This formulation provides the optimization target and feasibility boundary for the subsequent reinforcement learning-based power-control design.

## 3. Algorithm Design and Implementation

**Figure 2:**
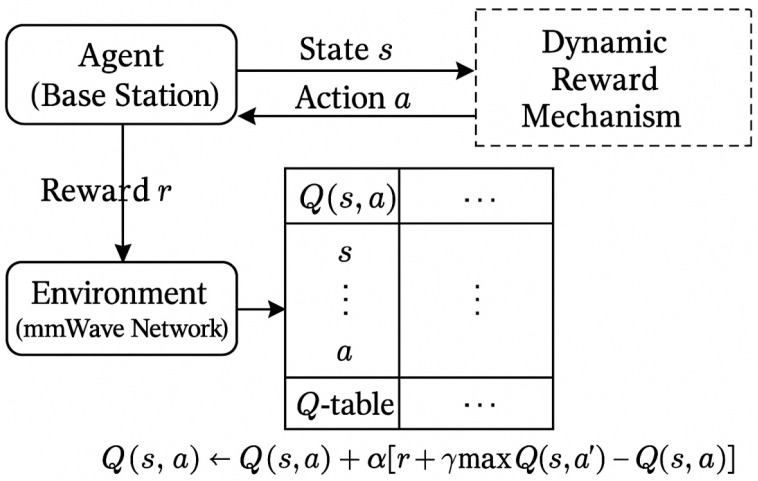
Q-learning-based power allocation framework with Q-table.

Figure 2 illustrates the interaction between each base station agent and the mmWave environment in the proposed distributed dynamic reward Q-learning framework. Each BS observes its local state, selects a power-adjustment action, receives immediate reward feedback from the environment, and updates its local Q-table according to the Q-learning update rule. The framework enables each BS to adapt its transmit power according to locally observable blockage, distance, and interference information without relying on a centralized controller.

### 3.1. Distributed Dynamic Reward Q-Learning Framework

In the proposed framework, each BS is modeled as an independent learning agent. Each agent observes local environmental information, including blockage state, user-distance level, and interference-intensity level, and selects a power-adjustment action from a finite action set. The agents do not share a centralized Q-table. Instead, each BS maintains its own Q-table and updates it using local state transitions and local reward feedback. The coupling among BS agents is reflected through the interference term, since the power decision of one BS affects the interference observed by neighboring BSs and users.

For BS i, the local state at time step t is defined as

(12) $$s_{i}\left(t\right)=\left[b_{i}\left(t\right),l_{i}^{d}\left(t\right),l_{i}^{I}\left(t\right)\right].$$

$$b_{i}\left(t\right)l_{i}^{d}\left(t\right)l_{i}^{I}\left(t\right)b_{i}\left(t\right)b_{i,k}\left(t\right)k\in U_{i}ib_{i}\left(t\right)=1b_{i}\left(t\right)=0b_{i}\left(t\right)=1\{{\left|U_{i}\right|}^{-1}\sum_{k\in U_{i}}b_{i,k}\left(t\right)\geq 0.5\}$$

where the first component denotes the discretized BS-level blockage ratio, the second component denotes the discretized average serving-distance level, and the third component denotes the discretized aggregate-interference level. For BS i, the blockage ratio is defined as the fraction of its associated users whose serving links are in the NLOS state. The ratio is quantized into three levels, allowing the state to distinguish limited, moderate, and widespread blockage. Majority-rule and worst-link encodings are retained only as comparison variants in Section 4.7; they are not used as the default state representation.

(13) $$l_{i}^{d}\left(t\right)\in \{1,2,\ldots ,L_{d}\},\;l_{i}^{I}\left(t\right)\in \{1,2,\ldots ,L_{I}\}.$$

$$l_{i}^{d}\left(t\right){\bar{d}}_{i}\left(t\right)={\left|U_{i}\right|}^{-1}\sum_{k\in U_{i}}d_{i,k}\left(t\right)l_{i}^{I}\left(t\right)2L_{d}L_{I}\left|A\right|L_{d}=3L_{I}=3\left|A\right|=32\times 3\times 3\times 3=54$$

The average serving distance is quantized using thresholds of 40 m and 80 m: near for *d_i_* < 40 m, medium for 40 m ≤ *d_i_* < 80 m, and far for *d_i_* ≥ 80 m. The aggregate interference is quantized in dBm using thresholds of −88 dBm and −76 dBm: low for I_i_ < −88 dBm, medium for −88 dBm ≤ I_i_ < −76 dBm, and high for I_i_ ≥ −76 dBm. These thresholds are fixed for all algorithms and all random seeds. They were selected to provide non-empty state occupancy across preliminary topology realizations. Section 4.7 additionally evaluates two to five quantization levels to assess the sensitivity of the tabular design.

For the power-control update, $p_{i}\left(t\right)$ denotes the dBm-scale transmit-power level, while $P_{i}\left(t\right)$ denotes the corresponding linear transmit power used in the SINR calculation, with $P_{i}\left(t\right)=10^{\left(p_{i}\left(t\right)-30\right)/10}$. The action space is defined as

(14) $$A=\left\{-\Delta p,0,+\Delta p\right\},$$

corresponding to the decreasing, maintaining, or increasing dBm-scale transmit-power level. After action $a_{i}\left(t\right)$ is selected, the dBm-scale transmit power is updated by

(15) $$p_{i}\left(t+1\right)=min\left\{p_{max},max\left\{p_{min},p_{i}\left(t\right)+a_{i}\left(t\right)\right\}\right\}.$$

The updated dBm-scale transmit-power level is then converted into the linear transmit power as $P_{i}\left(t+1\right)=10^{\left(p_{i}\left(t+1\right)-30\right)/10}$ for SINR calculation.

Following the classical tabular Q-learning algorithm proposed by Watkins and Dayan [8], the Q-value of each BS is updated as

(16) $$\begin{array}{c} Q_{i}(s_{i}(t),a_{i}(t))\leftarrow (1-\alpha )Q_{i}(s_{i}(t),a_{i}(t))\\ \;+\alpha \left[r_{i}(t)+\gamma \max_{a^{\prime }\in A}Q_{i}(s_{i}(t+1),a^{\prime })\right], \end{array}$$

where α is the learning rate, γ is the discount factor, and $r_{i}\left(t\right)$ is the immediate reward obtained after BS i takes action $a_{i}\left(t\right)$ in state $s_{i}\left(t\right)$. An ϵ-greedy policy is adopted. With probability ϵ, the agent randomly explores an action; otherwise, it selects the action with the highest current Q-value.

### 3.2. Dynamic Reward Mechanism

To improve the adaptability of the learning process under dynamic LOS/NLOS transitions, this paper introduces a dynamic reward mechanism. The immediate reward of BS i is defined as a weighted combination of normalized throughput reward, QoS satisfaction reward, interference penalty, transmit-power regularization, and blockage penalty:

(17) $$r_{i}\left(t\right)=w_{C}{\bar{C}}_{i}\left(t\right)+w_{Q}\rho_{i}\left(t\right)-w_{I}{\bar{I}}_{i}\left(t\right)-w_{P}{\bar{P}}_{i}\left(t\right)-w_{B}z_{i}^{NLOS}\left(t\right),$$

where the five weights in (17) are nonnegative condition-dependent coefficients. Let b_i_(t) ∈ [0,1] denote the local blockage ratio, q_i_(t) ∈ [0,1] the local QoS satisfaction ratio, and ĩ_i_(t) = clip((I_i_(t) + 100)/35,0,1) the normalized interference level. The coefficients used in the complete dynamic reward implementation are w_C_(t) = 0.44 + 0.10[1 − b_i_(t)], w_Q_(t) = 0.28 + 0.35[1 − q_i_(t)], w_I_(t) = 0.18 + 0.30ĩ_i_(t), w_P_(t) = 0.12, and w_B_(t) = 0.12 + 0.20b_i_(t). Thus, low QoS increases the emphasis on QoS recovery; high interference increases the interference penalty, and severe blockage increases the blockage penalty. The fixed-weight ablation uses w_C_ = 0.55, w_Q_ = 0.20, w_I_ = 0.06, w_P_ = 0.04, and w_B_ = 0.08. All reward components are normalized before combination.

(18) $${\bar{C}}_{i}\left(t\right)=\frac{\sum_{k\in U_{i}}C_{i,k}\left(t\right)}{C_{max}},$$

(19) $$\rho_{i}\left(t\right)=\frac{1}{\left|U_{i}\right|}\sum_{k\in U_{i}}q_{i,k}^{QoS}(t),$$

(20) $${\bar{I}}_{i}\left(t\right)=\frac{1}{I_{max}\left|U_{i}\right|}\sum_{k\in U_{i}}\sum_{j\neq i}P_{j}\left(t\right)G_{j,k}\left(t\right),\;{\bar{P}}_{i}\left(t\right)=\frac{P_{i}\left(t\right)}{P_{max}},$$

where $q_{i,k}^{QoS}(t)$ is the same binary QoS satisfaction indicator as defined in (9). Here, $C_{max}$ and $I_{max}$ are normalization constants used to keep the reward components on comparable scales.

This reward design encourages higher capacity and QoS satisfaction while penalizing excessive interference, unnecessary transmit power, and widespread blockage. Because the coefficient adaptation is deterministic and bounded, the reward scale remains controlled while its relative emphasis follows the current local network condition (Algorithm 1).
**Algorithm 1:** Distributed dynamic reward Q-learning for blockage-aware power allocationInput: BS set, B; dBm-scale transmit-power limits, $p_{min}$ and $p_{max}$; power-adjustment step, Δp; learning rate, α; discount factor, γ; exploration probability, ϵ; number of episodes, E; number of time steps per episode, T. Output: Distributed power-allocation policy for each BS.
        1. Initialize the local Q-table $Q_{i}\left(s_{i},a_{i}\right)$ for each BS i∈B.
        2. Initialize the dBm-scale transmit-power level $p_{i}\left(0\right)$ for each BS.
        For episode e=1,…,E,
                3. Initialize the environment and local states.
                4. For time step t=0,…,T−1,
                        5. Observe the local state $s_{i}\left(t\right)$ for each BS i.
                        6. Select action $a_{i}\left(t\right)\in A$ according to the ϵ-greedy policy.
                        7. Update the dBm-scale transmit-power level according to (15).
                        8. Convert the updated dBm-scale power to the linear power $P_{i}\left(t+1\right)=10^{\left(p_{i}\left(t+1\right)-30\right)/10}$.
                        9. Compute SINR, local capacity, QoS satisfaction ratio, interference level, and blockage indicator.
                        10. Compute the dynamic reward $r_{i}\left(t\right)$.
                        11. Observe the next local state $s_{i}\left(t+1\right)$.                        12. Update $Q_{i}\left(s_{i}\left(t\right),a_{i}\left(t\right)\right)$ according to (16).
                13. End for
        14. End for
        15. Return the learned distributed power-allocation policy.

### 3.3. DQN-Based Baseline

To provide a stronger learning-based comparison, a DQN-based power-allocation baseline is implemented using the same local state variables, action set, dynamic reward, training episodes, evaluation realizations, and exploration schedule as the proposed method. The DQN uses a three-input, one-hidden-layer neural network with 24 ReLU units and three action outputs, together with experience replay and a target network. The final learning rate, replay size, batch size, and target-update interval were selected from a predefined lightweight validation grid using seeds excluded from the reported test runs. The tested values were learning rate {3 × 10^−4^, 8 × 10^−4^, 10^−3^}, hidden width {16, 24, 32}, batch size {32, 48, 64}, and target-update interval {60, 90, 120} steps. The selected configuration is reported in Table 1. This tuning procedure improves fairness, although the DQN remains a compact comparison model rather than an exhaustively optimized upper bound.

### 3.4. Complexity Analysis

For the proposed tabular dynamic reward Q-learning method, the storage complexity of each BS is $O\left(L_{b}L_{d}L_{I}\left|A\right|\right)$, where $L_{b}$, $L_{d}$, and $L_{I}$ are the numbers of blockage-ratio, distance, and interference levels, respectively. With three levels for each state component and three actions, each BS stores 3 × 3 × 3 × 3 = 81 Q-values. Greedy action selection requires $O\left(\left|A\right|\right)$ comparisons, and a single Q-table update is $O\left(1\right)$. The DQN baseline requires a neural-network forward pass for action selection and back-propagation during training; with three inputs, 24 hidden units, and three outputs, it contains 171 trainable scalar parameters. These computational quantities are complemented by the communication-overhead discussion in Section 4.10.

## 4. Simulation Results

### 4.1. Experimental Protocol and Parameter Configuration

Because the original source code was not available, all experiments were independently reimplemented from the system model, algorithm definitions, and parameter descriptions reported in the manuscript. The same reconstructed simulator was used for every compared method. For each random seed, the algorithms were evaluated using identical BS and UE locations, user-mobility traces, LOS/NLOS transitions, shadow-fading realizations, directional-gain realizations, and evaluation time steps. This paired design reduces variability caused by the environment and enables direct algorithm-level comparison. The implementation is therefore reproducible, although it should not be interpreted as a bit-for-bit reconstruction of the unavailable original program.

Table 1 lists the common physical-layer, mobility, blockage, learning, and DQN settings. Unless otherwise stated, the primary blockage sweep uses 30 paired Monte Carlo runs. The ablation, state-representation, quantization, and beam-misalignment studies use 10 paired runs to limit computational cost while preserving a common-seed comparison. Training and evaluation are separated: learned policies are trained for the specified episode budget and are then evaluated on paired environment realizations without altering the baseline definitions.

### 4.2. Evaluation Metrics and Statistical Procedure

Performance is evaluated using average system capacity, QoS satisfaction rate, average transmit power, and learning-curve behavior. System capacity is obtained by summing the instantaneous user rates defined in Section 2, whereas QoS satisfaction is the proportion of users whose SINR is not lower than the prescribed threshold. For a sample mean $\bar{x}$ obtained from n independent paired runs, the reported 95% confidence-interval half-width is 1.96 s/√n, where s denotes the sample standard deviation. At the severe-blockage operating point p_b_ = 0.5, paired t-tests compare the proposed method with each baseline for both capacity and QoS. The eight resulting *p*-values are adjusted by the Holm procedure, and Cohen’s d_z_ is reported as the paired mean difference divided by the standard deviation of the paired differences. This procedure distinguishes numerical differences from statistically supported differences.

### 4.3. Main Performance Under Varying Blockage Probabilities

#### 4.3.1. System Capacity

**Figure 3:**
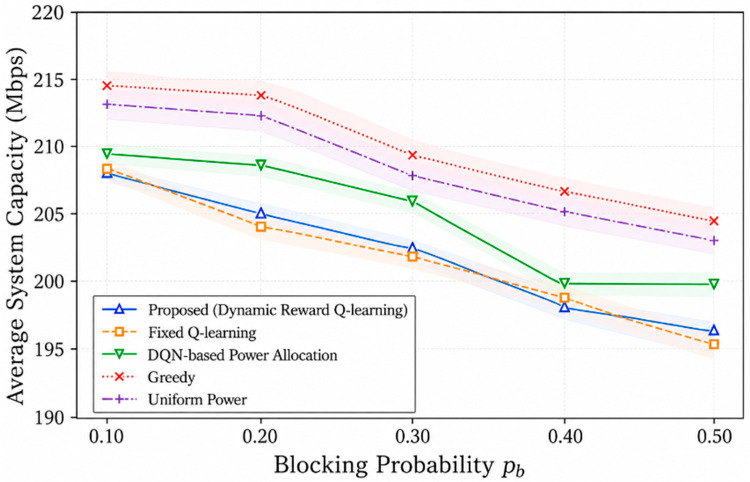
Average system capacity versus blockage probability for different power allocation strategies.

Figure 3 shows that the average system capacity decreases as the blockage probability increases. This trend is consistent with the channel model: a larger p_b_ causes more links to operate under the NLOS path-loss exponent and the larger NLOS shadow-fading variance. Across the blockage sweep, the proposed dynamic reward method and fixed-weight Q-learning remain close, indicating that condition-dependent coefficient adaptation produces only a limited throughput change in the reconstructed four-BS scenario. DQN gives moderately higher capacity at several blockage levels, whereas greedy and uniform power attain the largest raw throughput. The latter result is explained by the relatively small network, weak directional-interference coupling, and absence of a hard average-power or energy-efficiency constraint in the capacity objective. Consequently, these curves should be interpreted as a throughput comparison under the stated assumptions rather than evidence that high-power operation is generally preferable.

#### 4.3.2. QoS Satisfaction

**Figure 4:**
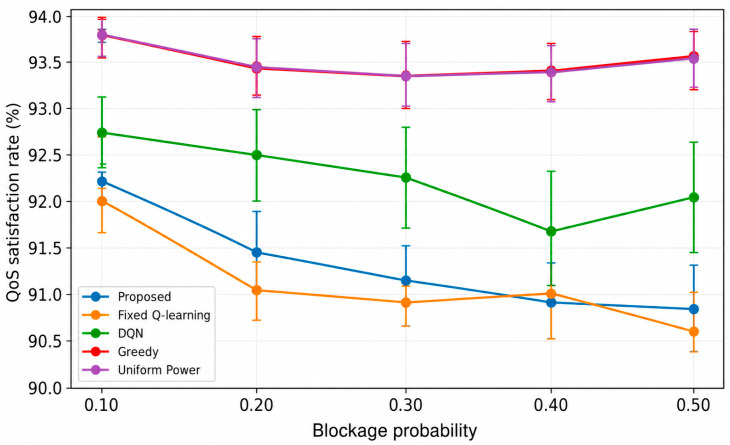
QoS satisfaction rate versus blockage probability. Error bars show 95% confidence intervals over 30 paired runs.

Figure 4 presents the QoS satisfaction rate over the same blockage sweep. The ordering broadly follows the capacity results because a higher received signal level increases both rate and the probability of satisfying the SINR threshold. At p_b_ = 0.5, the proposed method achieves 90.85% QoS satisfaction, compared with 90.61% for fixed-reward Q-learning, 92.07% for DQN, 93.62% for greedy, and 93.59% for uniform power. The comparatively high absolute values arise from the 1 MHz bandwidth, the resulting low receiver-noise power, and the 34 dB aligned desired-link gain. These favorable values do not imply immunity to directional errors: Section 4.8 shows that deterioration of the desired beam gain causes a much larger QoS and capacity loss than the reward-design variations examined here.

### 4.4. Statistical Comparison Under Severe Blockage

Table 2 and Table 3 summarize the paired 30-run results at p_b_ = 0.5. The capacity and QoS differences between the proposed method and fixed-reward Q-learning are not statistically significant after Holm correction (adjusted *p* = 0.421 for both metrics). The proposed method has a QoS rate 1.22 percentage points lower than DQN, with an adjusted *p*-value of 0.0227, whereas the corresponding capacity difference is not significant after correction (adjusted *p* = 0.103). Greedy and uniform power significantly exceed the proposed method in raw capacity and QoS under the reconstructed assumptions. Accordingly, the revised interpretation is deliberately limited: the proposed design provides a low-complexity, locally observable control policy and a transparent multi-objective reward formulation, but the present experiment does not establish universal throughput superiority over all baselines.

### 4.5. Learning Dynamics and Empirical Convergence

**Figure 5:**
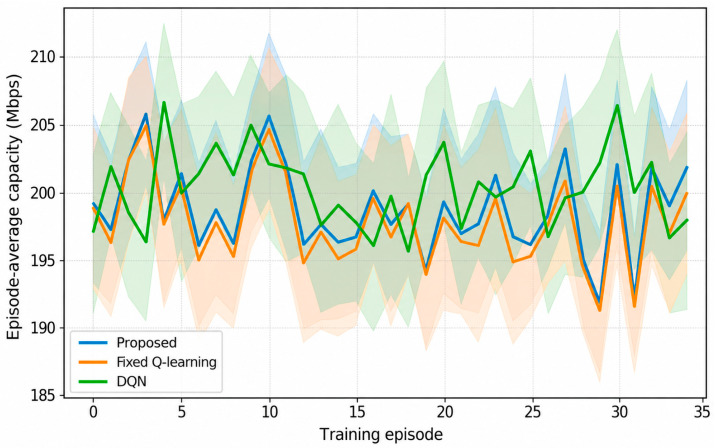
Episode-average capacity of the three learning-based methods at p_b_ = 0.5. Shaded regions show 95% confidence intervals over 30 runs.

Figure 5 reports episode-average capacity for the three learning-based methods at p_b_ = 0.5. The curves fluctuate rather than increase monotonically because user positions, blockage states, shadow fading, directional gains, and the policies of neighboring independent agents all change during training. The tabular methods enter a broad performance plateau within the 35-episode budget and require only table lookup and update operations. DQN exhibits additional variability due to stochastic replay sampling, function approximation, and target-network updates. Therefore, the figure is interpreted as evidence of empirical policy stabilization under the adopted finite training budget; it is not presented as a proof of convergence to a globally optimal joint multi-agent policy.

### 4.6. Reward-Component Ablation

**Figure 6:**
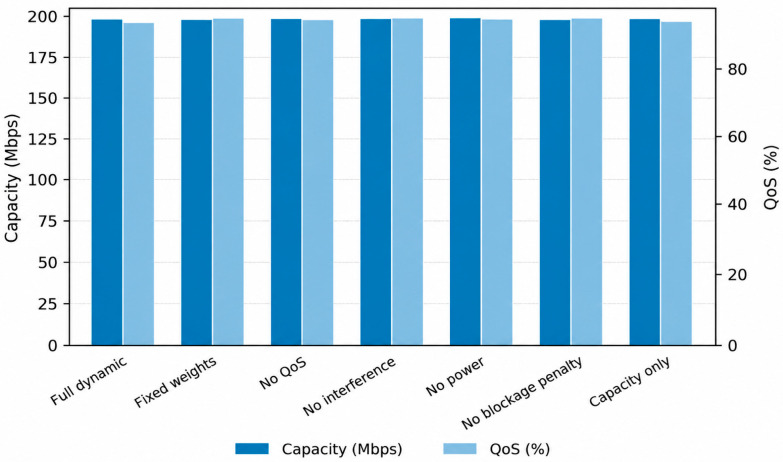
Reward-component ablation at p_b_ = 0.5 using 10 paired runs.

Figure 6 and Table 4 isolate the effect of replacing the condition-dependent coefficients with fixed coefficients and the effects of removing the QoS, interference, power, and blockage terms. The 10-run confidence intervals overlap substantially, and the mean-capacity change in every ablated variant is below 1 Mbps relative to the complete reward. Fixed coefficients produce nearly the same capacity and slightly higher mean QoS, whereas the complete dynamic reward uses approximately 1 dB more in average transmit power. Therefore, the current experiment supports the interpretability and reproducibility of the multi-component reward, but it does not establish that coefficient adaptation is independently responsible for a statistically significant performance gain in this topology.

### 4.7. State Representation and Quantization Sensitivity

**Figure 7:**
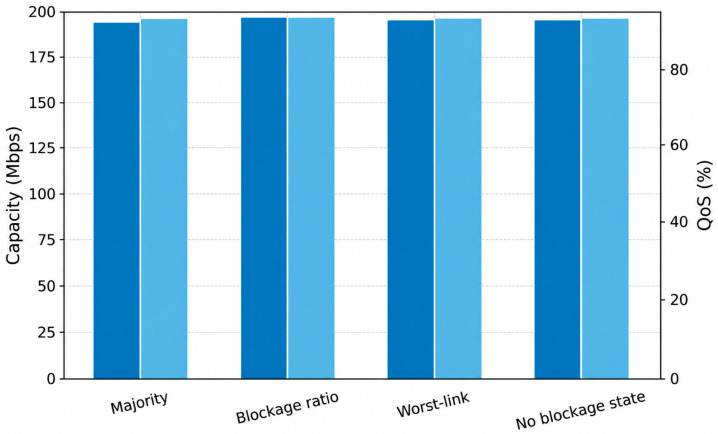
Comparison of blockage-ratio, majority, worst-link, and no-blockage state representations at p_b_ = 0.5 (10 paired runs).

Figure 7 compares four blockage-state designs at p_b_ = 0.5. The blockage-ratio representation achieves 199.18 Mbps, while majority aggregation, worst-link aggregation, and omission of the blockage component yield 197.59, 198.84, and 198.80 Mbps, respectively. The associated QoS values are also close. The ratio state is retained because it distinguishes partial blockage from widespread blockage and avoids the information loss of a single majority bit. However, the small numerical separation and overlapping uncertainty do not justify claiming a large statistically established advantage in the current topology.

**Figure 8:**
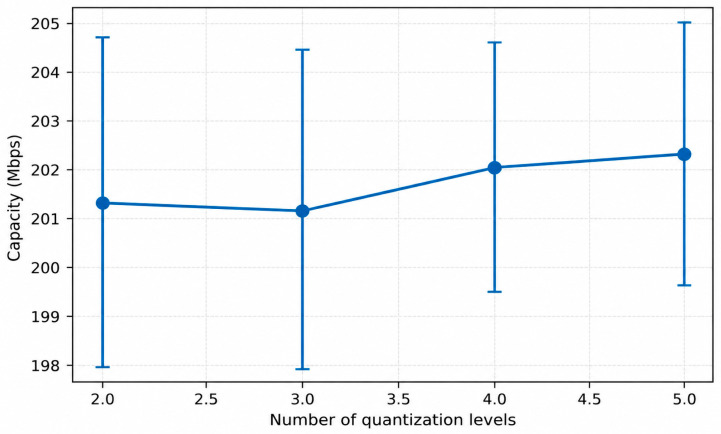
Capacity sensitivity to two, three, four, and five quantization levels at p_b_ = 0.5 (10 paired runs).

Figure 8 evaluates two, three, four, and five quantization levels. Mean capacity ranges from 201.17 to 202.35 Mbps for three to five levels, while the two-level design gives 201.33 Mbps. The performance difference is small relative to the confidence intervals, but the storage cost grows quickly. With L levels for each of the three state components and three actions, each BS stores 3L^3^ Q-values: 24 values for L = 2, 81 for L = 3, 192 for L = 4, and 375 for L = 5. Three levels are therefore adopted as a practical balance between state resolution and table size rather than as a uniquely optimal setting.

### 4.8. Beam-Misalignment Robustness

**Figure 9:**
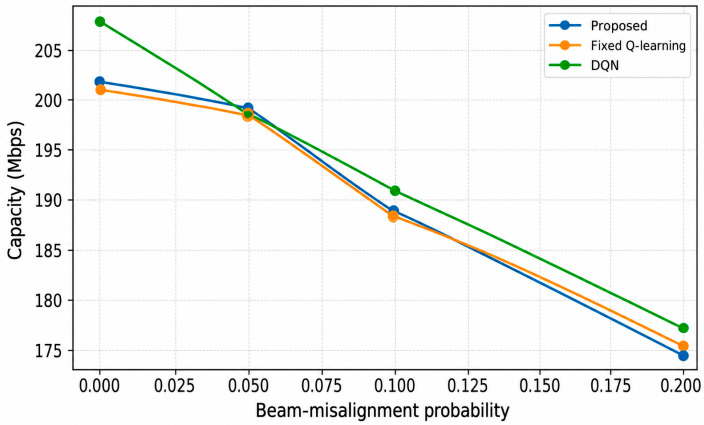
Capacity under stochastic desired-beam misalignment at p_b_ = 0.5 (10 paired runs).

Figure 9 evaluates stochastic desired-beam misalignment and directly addresses the limitation of fixed aligned antenna gains. When the misalignment probability increases from 0 to 0.20, the proposed method’s capacity decreases from 201.74 to 174.94 Mbps, while its QoS satisfaction rate falls from 92.79% to 84.08%. At p_mis_ = 0.20, fixed-reward Q-learning and DQN achieve 175.78 and 177.45 Mbps, respectively. All three methods exhibit similar degradation, showing that transmit-power control alone cannot compensate for a severe loss of directional array gain. The result supports treating beam alignment as a separate but coupled control process and motivates future joint beam-tracking and power-allocation research.

### 4.9. Reproducibility and Scope of Interpretation

The accompanying reproducibility package contains the complete Python implementation, primary raw CSV data, statistical summaries, and figure-generation scripts. All experiments were conducted using Python 3.13.5, with NumPy 2.3.5, Pandas 2.2.3, SciPy 1.17.0, Matplotlib 3.10.8, and PyTorch 2.10.0 (CPU version). Fixed seed schedules and paired environment realizations were used throughout. The reported findings therefore provide verifiable evidence for the reconstructed model and parameter set. They do not recover the unavailable original code, validate unsupported numerical values from an earlier draft, or establish superiority for arbitrary network densities, bandwidths, antenna arrays, mobility patterns, traffic loads, or beam-management procedures. This scope limitation is carried forward explicitly into the conclusion.

### 4.10. Communication and Computational Overhead

A centralized controller would require network-wide reports of serving and interfering channel information before distributing a joint power vector. With N_BS_ base stations and N_UE_ users, a direct reporting model requires O(N_BS_ N_UE_) channel or interference quantities per decision interval, in addition to N_BS_ downlink control commands. In the proposed distributed method, each BS receives only local UE measurements required to form its blockage ratio, average serving distance, and aggregate-interference level. No Q-table, intended action, or per-link global CSI is exchanged among BSs. The inter-BS signaling requirement is therefore zero in the adopted implementation, while local feedback scales as O(N_UE,i_) per BS. Online computation consists of |A| = 3 Q-value comparisons and one scalar Q update per BS and time step at most; storage is 81 Q-values per BS. By comparison, the DQN uses the same local feedback but requires a 171-parameter neural-network forward pass online and replay-based back-propagation during training. Control delay is not reported in milliseconds because no hardware or transport-network latency model was measured; the comparison is stated in reproducible message-count and operation-count terms rather than unsupported timing units.

## 5. Conclusions and Future Work

This paper developed a distributed blockage-aware power-control framework for dynamic millimeter-wave communication networks using state-dependent dynamic reward Q-learning. The system model incorporates clustered users, fixed user association within each episode, user mobility, temporal LOS/NLOS evolution, 3GPP-style path loss, shadow fading, directional antenna gains, stochastic beam misalignment, coupled inter-base station interference, SINR, system capacity, and QoS satisfaction. Each BS operates as an independent learning agent and selects a discrete transmit-power adjustment using a compact local state composed of blockage ratio, serving-distance level, and aggregate-interference level. The reward coefficients adapt deterministically to the instantaneous blockage ratio, QoS satisfaction, and interference condition, while the tabular implementation retains low storage and online computational complexity.

The independently reconstructed experiments provide a cautious and reproducible assessment of the method. At a blockage probability of 0.5, the proposed algorithm achieves an average system capacity of 196.69 Mbps, a QoS satisfaction rate of 90.85%, and an average transmit power of 20.14 dBm. Its capacity and QoS are statistically indistinguishable from those of fixed-weight Q-learning after Holm correction. Under the same reconstructed setting, DQN achieves 200.25 Mbps and 92.07% QoS satisfaction, while greedy and uniform power obtain higher raw capacity and QoS in the relatively small and weak interference-coupled network. The ablation and state-representation studies show only modest differences among the tested reward components and local blockage encodings, whereas the beam-misalignment analysis reveals a substantial capacity reduction as directional gain is lost. The proposed method therefore offers a transparent, locally observable, and low-complexity control design, but the present results do not establish universal performance superiority.

Several limitations should be acknowledged. The simulator is an independent reconstruction because the original implementation was unavailable. User association is fixed during each episode, and handover, load balancing, and traffic variation are not modeled. The temporal blockage process is Markov-based rather than generated by explicit blocker geometry. Beam misalignment is represented through effective antenna-gain events and does not fully capture array geometry, side-lobe structure, beam codebooks, training overhead, or tracking delay. In addition, simultaneous independent learners experience a non-stationary environment, so no guarantee of convergence to a globally optimal joint policy is claimed. The evaluated network contains four BSs and sixteen UEs, and the discrete state-action design may not scale directly to larger continuous deployments. The DQN baseline is deliberately lightweight; the secondary analyses use 10 paired runs, and all findings are simulation-based. No measured control-delay claim is made.

Future work will extend the framework in several directions. First, joint beam selection, beam tracking, and power allocation should be formulated to account explicitly for directional antenna dynamics. Recent deep reinforcement learning studies have shown that dynamic beam selection can reduce beam-sweeping overhead while preserving link quality [15], whereas sequence-learning models can exploit temporal channel correlation for selective multi-cell beam tracking [16]. Second, geometry-based blockage, adaptive association, handover, and load balancing should be integrated into the state-transition model. Multimodal sensing, including location and environmental information, may further improve blockage prediction and multi-user beamforming decisions [17]. Third, interference-aware online beam learning offers a promising way to shape practical mmWave beams without requiring explicit global channel knowledge [18]. Finally, the present distributed power-control formulation can be extended to joint user clustering, beamforming, and power allocation, where inter-cluster and intra-cluster interferences are optimized together under QoS constraints [19]. These extensions should be evaluated using larger network layouts, standardized channel traces, hardware-in-the-loop experiments, and measured mmWave datasets.

## Figures and Tables

**Table 1:** Main simulation and learning parameters.

Category	Setting
Network	4 BSs; 4 UEs per BS; 200 m × 200 m area; Gaussian cluster spread 25 m
Carrier and bandwidth	28 GHz; 1 MHz
Noise	−174 dBm/Hz thermal density; 7 dB receiver noise figure
Power	10–30 dBm; 2 dB action step; initial/uniform power 20 dBm
Channel	3GPP-style UMi LOS/NLOS path loss; shadowing σ_LOS_ = 4 dB, σ_NLOS_ = 7.8 dB
Blockage	p_b_ ∈ {0.1, 0.2, 0.3, 0.4, 0.5}; temporal update probability 0.08
Directional gains	Desired aligned 34 dB; misaligned 5 dB; interference gains 20/10/2 dB
Mobility	Bounded random walk, 1 m per decision step
QoS	SINR threshold 10 dB
Q-learning	α = 0.18; γ = 0.92; ε: 0.90 → 0.05; decay 0.965
Training	35 episodes × 15 steps; 8 evaluation episodes; 30 paired primary runs
DQN	3 inputs; 24 ReLU hidden units; 3 outputs; replay 5000; batch 48; target update 90 steps; lr 8 × 10^−4^
Secondary analyses	10 paired runs for ablation, state representation, quantization, and beam misalignment
State discretization	Blockage ratio: 3 equal-width levels; distance thresholds: 40/80 m; interference thresholds: −88/−76 dBm
Dynamic reward coefficients	w_C_ = 0.44 + 0.10(1 − b); w_Q_ = 0.28 + 0.35(1 − q); w_I_ = 0.18 + 0.30ĩ; w_P_ = 0.12; w_B_ = 0.12 + 0.20b
DQN validation grid	lr {3 × 10^−4^, 8 × 10^−4^, 10^−3^}; hidden {16, 24, 32}; batch {32, 48, 64}; target update {60, 90, 120}

**Table 2:** Primary results at blockage probability p_b_ = 0.5 (30 paired runs).

Method	Capacity Mean ± SD (Mbps)	95% CI Half-Width	QoS Mean ± SD (%)	95% CI Half-Width	Avg. Power (dBm)
Proposed	196.69 ± 6.05	±2.17	90.85 ± 1.31	±0.47	20.14
Fixed Q-learning	196.30 ± 5.67	±2.03	90.61 ± 1.24	±0.44	20.04
DQN	200.25 ± 6.82	±2.44	92.07 ± 1.76	±0.63	19.71
Greedy	204.57 ± 5.77	±2.06	93.62 ± 1.04	±0.37	30.00
Uniform Power	203.36 ± 5.71	±2.04	93.59 ± 1.05	±0.37	20.00

**Table 3:** Paired comparisons of the proposed method against baselines at p_b_ = 0.5.

Metric	Baseline	Proposed—Baseline	Raw p	Holm-Adjusted p	Cohen d_z_
Capacity	Fixed Q-learning	0.383 Mbps	0.3885	0.4209	0.160
QoS	Fixed Q-learning	0.233 percentage points	0.2105	0.4209	0.234
Capacity	DQN	−3.568 Mbps	0.03445	0.1034	−0.405
QoS	DQN	−1.219 percentage points	0.005669	0.02267	−0.545
Capacity	Greedy	−7.881 Mbps	2.383 × 10^−5^	0.000143	−0.917
QoS	Greedy	−2.774 percentage points	6.571 × 10^−9^	5.257 × 10^−8^	−1.475
Capacity	Uniform Power	−6.674 Mbps	0.0001901	0.0009504	−0.780
QoS	Uniform Power	−2.747 percentage points	8.671 × 10^−9^	6.069 × 10^−8^	−1.455

**Table 4:** Reward-component ablation at p_b_ = 0.5.

Variant	Capacity Mean ± 95% CI (Mbps)	QoS Mean ± 95% CI (%)	Avg. Power (dBm)
Complete dynamic reward	198.94 ± 4.71	91.43 ± 1.13	20.42
Fixed coefficients	198.91 ± 4.75	92.01 ± 0.99	19.40
Without QoS term	199.31 ± 4.15	92.02 ± 0.98	20.24
Without interference term	199.16 ± 4.36	92.08 ± 0.69	18.75
Without power term	199.62 ± 4.35	91.93 ± 0.66	19.44
Without blockage penalty	198.88 ± 3.63	92.10 ± 0.57	19.28
Capacity-only reward	199.12 ± 3.98	91.60 ± 0.67	19.90

## Data Availability

The simulation data used in this study are available from the corresponding author upon reasonable request.
